# Supplementary material for: Incidence and predictors of tuberculosis among HIV-infected children after initiation of antiretroviral therapy in Ethiopia: A systematic review and meta-analysis
Source: PLoS One. 2024 Jul 5;19(7):e0306651. doi: 10.1371/journal.pone.0306651 (PMC11226042; doi:10.1371/journal.pone.0306651)
Supplement: S2 Checklist — (DOCX) [file pone.0306651.s002.docx]

# JBI critical appraisal checklist for cohort studies

| **Primary studies** |  | **JBI’s critical appraisal questions** | | | | | | | | | | Overall quality score (%) | Included |
| --- | --- | --- | --- | --- | --- | --- | --- | --- | --- | --- | --- | --- | --- |
|  | Q1 | Q2 | Q3 | Q4 | Q5 | Q6 | Q7 | Q8 | Q9 | Q10 | Q11 |  |  |
| (Alemu et al., 2016) | Y | Y | Y | Y | N | Y | Y | y | N | Y | Y | 90.9 | √ |
| (Ayalaw et al., 2015) | Y | Y | Y | Y | N | Y | Y | Y | N | Y | Y | 82 | √ |
| (Beshir et al., 2019) | y | Y | Y | Y | N | Y | Y | N | N | Y | Y | 82 | √ |
| (Endalamaw et al., 2018) | Y | Y | Y | Y | N | Y | Y | Y | Y | Y | Y | 90.9 | √ |
| (Jerene et al., 2017) | Y | Y | Y | Y | N | Y | Y | Y | Y | N | Y | 90.9 | √ |
| (Kebede et al., 2021) | Y | Y | Y | Y | N | Y | Y | Y | N | Y | Y | 82 | √ |
| (Kebede et al., 2022) | Y | Y | Y | Y | N | Y | Y | Y | N | Y | Y | 82 | √ |
| (Tekese et al., 2023) | Y | Y | Y | Y | Y | Y | Y | Y | N | N | Y | 82 | √ |
| (Tsegaye et al., 2023) | Y | Y | Y | Y | N | Y | Y | Y | Y | N | Y | 82 | √ |
| (Wondifraw et al., 2022) | Y | Y | Y | Y | N | Y | Y | Y | N | Y | Y | 82 | √ |

Q1: Were the two groups similar and recruited from the same population?

Q2: Were the exposures measured similarly to assign people to both exposed and unexposed (Marie et al., 2022)groups?

Q3: Was the exposure measured in a valid and reliable way?

Q4: Were confounding factors identified?

Q5: Were strategies to deal with confounding factors stated?

Q6: Were the groups/participants free of the outcome at the start of the study (or at the moment of exposure)?

Q7: Were the outcomes measured in a valid and reliable way?

Q8: Was the follow up time reported and sufficient to be long enough for outcomes to occur?

Q9: Was follow up complete, and if not, were the reasons to loss to follow up described and explored?

Q10: Were strategies to address incomplete follow up utilized?

Q11: Was appropriate statistical analysis used?

**NB**: Y: Yes, N: No, U: Unclear, Q: Question. The overall score is calculated by counting the number of Y’s in each row.
