# Supplementary figures and images for: Incidence and predictors of tuberculosis among HIV-infected children after initiation of antiretroviral therapy in Ethiopia: A systematic review and meta-analysis
Source: PLoS One. 2024 Jul 5;19(7):e0306651. doi: 10.1371/journal.pone.0306651 (PMC11226042; doi:10.1371/journal.pone.0306651)

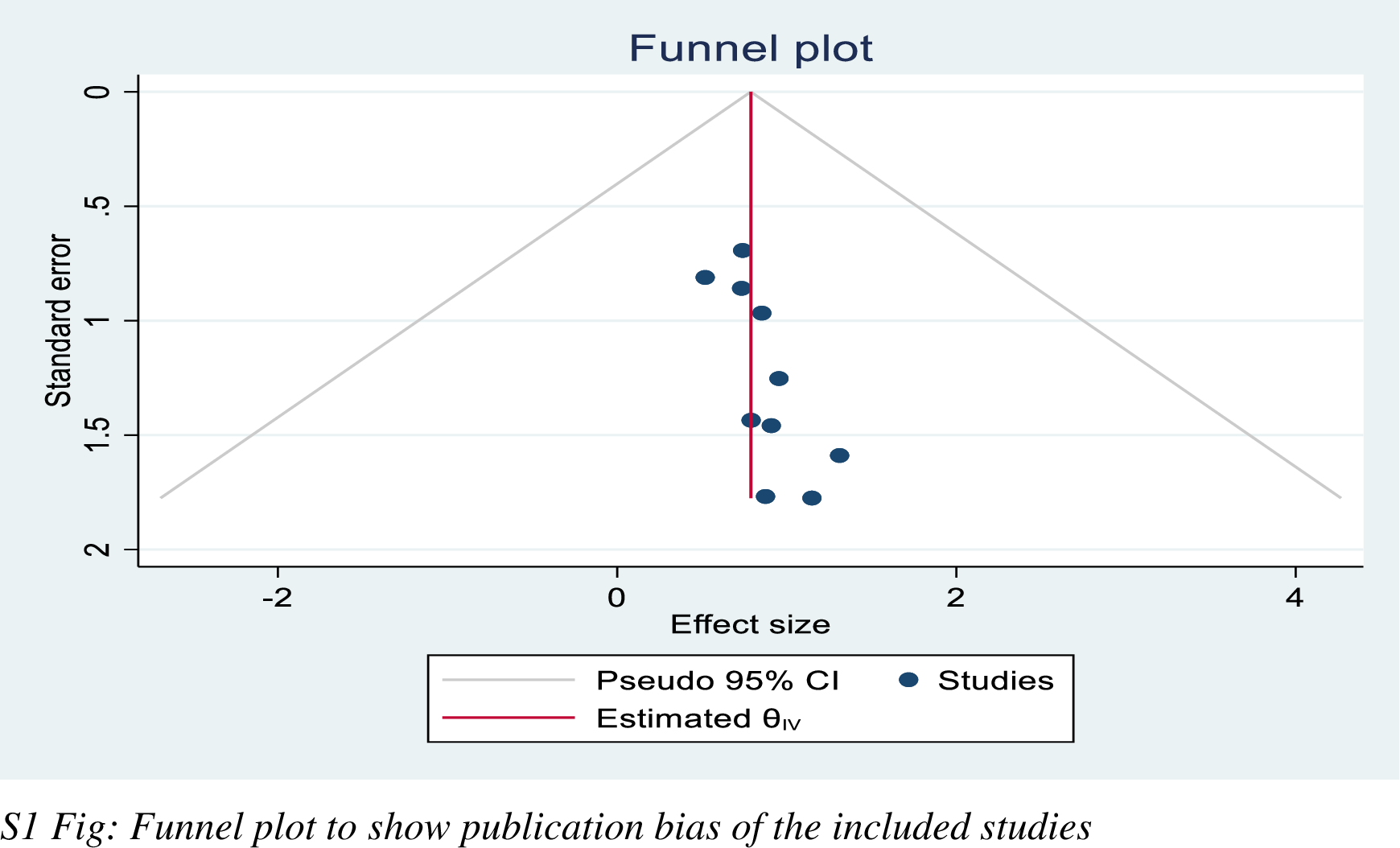

Supplement: S1 Fig — (TIF) [file pone.0306651.s003.tif]

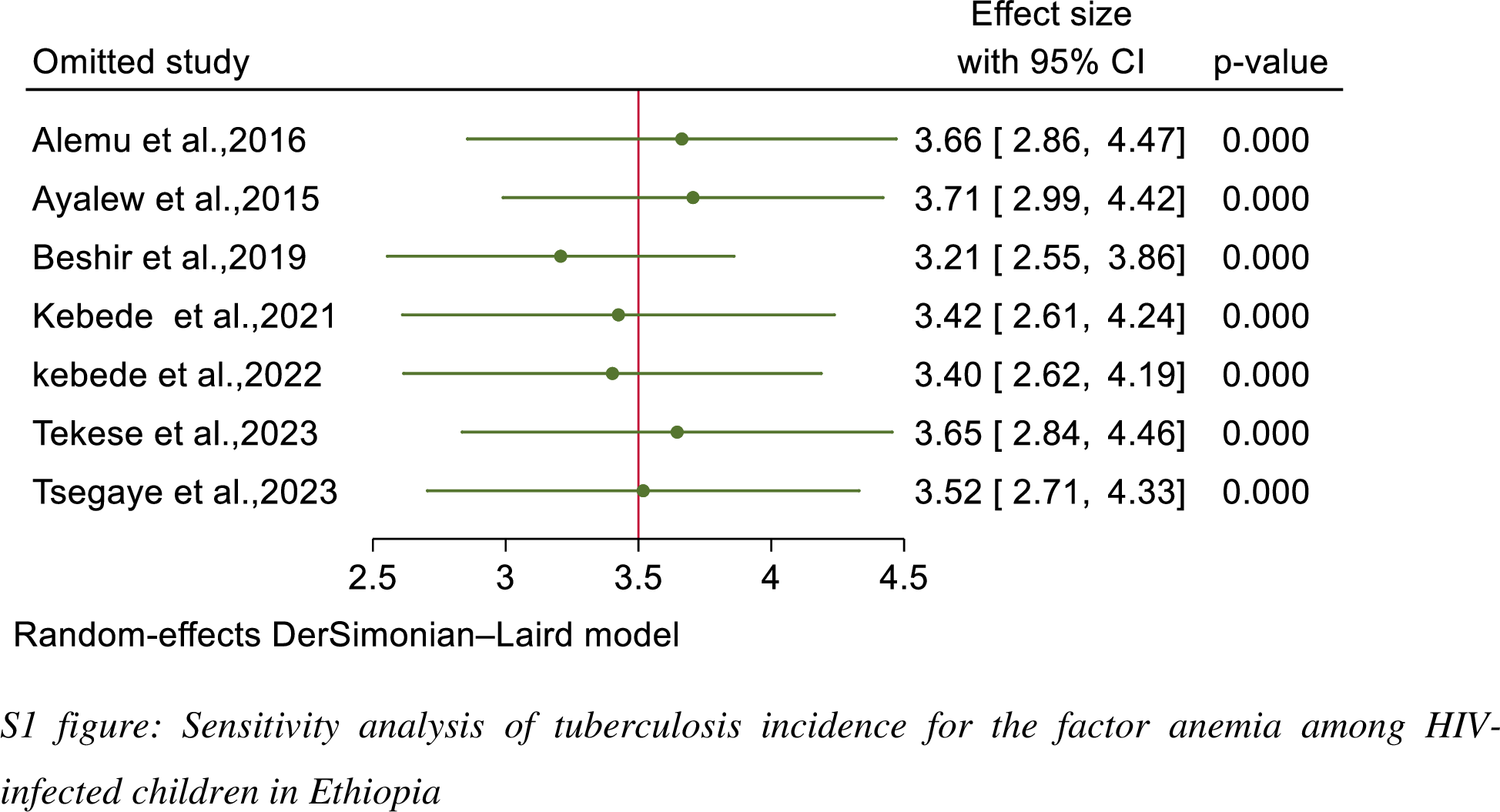

Supplement: S2 Fig — (TIF) [file pone.0306651.s004.tif]

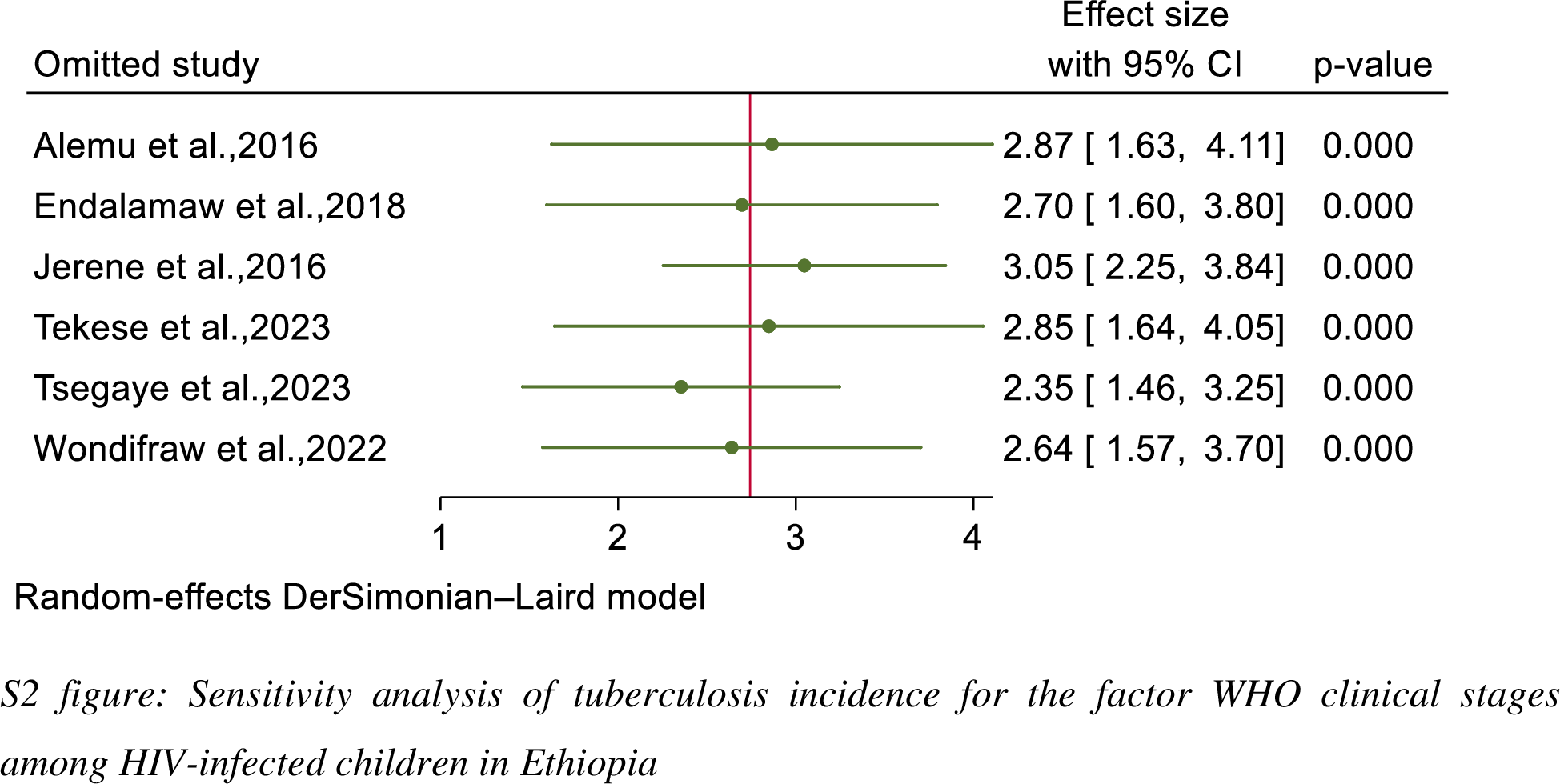

Supplement: S3 Fig — (TIF) [file pone.0306651.s005.tif]
