# Supplementary material for: Incidence and predictors of tuberculosis among HIV-infected children after initiation of antiretroviral therapy in Ethiopia: A systematic review and meta-analysis
Source: PLoS One. 2024 Jul 5;19(7):e0306651. doi: 10.1371/journal.pone.0306651 (PMC11226042; doi:10.1371/journal.pone.0306651)
Supplement: S1 Table — (DOCX) [file pone.0306651.s006.docx]

| S1 table: Studies search strategies and entry terms from different electronic data bases on **Incidence and predictors of tuberculosis among HIV-infected children after initiation of antiretroviral therapy in Ethiopia** | |
| --- | --- |
| Search in each key words and Mesh terms | **Pumed/MEDLINE database** |
| **#1** | "Incidence"[MeSH] OR Incidence OR proportions OR "incidence rate" OR "incidence density" |
| **#2** | predictors OR associated factors OR determinants OR risk factors |
| **#3** | "Tuberculosis"[MeSH] OR Tuberculosis OR "pulmonary tuberculosis" |
| **#4** | "HIV Infections"[Mesh] OR "HIV-infection" OR "HIV-positive children" OR "HIV- infected children" |
| **#5** | "Child"[MeSH] OR child* OR pediatric* OR paediatric* |
| **#6** | "Antiretroviral Therapy, Highly Active"[MeSH] OR "anti-retroviral agents" OR "anti-retroviral agents"[MeSH] OR "antiretroviral treatment" |
| **#7** | Ethiopia |
| **Final** | "Incidence"[MeSH] OR Incidence OR proportions OR "incidence rate" OR "incidence density" AND predictors OR associated factors OR determinants OR risk factors AND "Tuberculosis"[MeSH] OR Tuberculosis OR "pulmonary tuberculosis" AND "HIV Infections"[Mesh] OR "HIV-infection" OR "HIV-positive children" OR "HIV- infected children" AND "Child"[MeSH] OR child* OR pediatric* OR paediatric* AND "Antiretroviral Therapy, Highly Active"[MeSH] OR "anti-retroviral agents" OR "anti-retroviral agents"[MeSH] OR "antiretroviral treatment" AND Ethiopia AND ((ffrft[Filter]) AND (humans[Filter]) AND (English[Filter]) AND (2014:2024[pdat])) =**476** |
| **Search results** | **476 free full text articles from 2014-2024** |
| **Search date** | **January 5/2024** |
|  | **Google scholar database** |
| With all of the words | Incidence AND predictors AND Tuberculosis AND "HIV-infection" AND OR children AND “Antiretroviral Therapy” AND Ethiopia |
| With exact phrases | Tuberculosis |
| with **at least one** of the words | (Predictors OR associated factors OR determinants) |
| **Final Search builds** | Incidence AND predictors AND Tuberculosis AND "HIV-infection" AND OR children AND “Antiretroviral Therapy” AND Ethiopia AND Tuberculosis AND (Predictors OR associated factors OR determinants)=**279** |
| **Total search results** | **279 articles from 2014-2024** |
| **Search date** | **January 10/2024** |
