# Supplementary material for: Incidence and predictors of tuberculosis among HIV-infected children after initiation of antiretroviral therapy in Ethiopia: A systematic review and meta-analysis
Source: PLoS One. 2024 Jul 5;19(7):e0306651. doi: 10.1371/journal.pone.0306651 (PMC11226042; doi:10.1371/journal.pone.0306651)
Supplement: Attachment — Submitted filename: Feedback_Amare_childhhod TB.docx [file pone.0306651.s008.docx]

Academic Editor

Congratulation again to come up with this very relevant manuscript of childhood TB among HIV infected children on ART in Ethiopia. It is well narrated, reviewed, analyzed and discussed. Please just attend to a few comments from the one of the revue

Regards

Reviewers' comments:

Reviewer's Responses to Questions

**Comments to the Author**

1. Is the manuscript technically sound, and do the data support the conclusions?

The manuscript must describe a technically sound piece of scientific research with data that supports the conclusions. Experiments must have been conducted rigorously, with appropriate controls, replication, and sample sizes. The conclusions must be drawn appropriately based on the data presented.

Reviewer #1: Yes

Reviewer #2: Yes

2. Has the statistical analysis been performed appropriately and rigorously?

Reviewer #1: Yes

Reviewer #2: Yes

3. Have the authors made all data underlying the findings in their manuscript fully available?

The [PLOS Data policy](http://www.plosone.org/static/policies.action#sharing) requires authors to make all data underlying the findings described in their manuscript fully available without restriction, with rare exception (please refer to the Data Availability Statement in the manuscript PDF file). The data should be provided as part of the manuscript or its supporting information, or deposited to a public repository. For example, in addition to summary statistics, the data points behind means, medians and variance measures should be available. If there are restrictions on publicly sharing data—e.g. participant privacy or use of data from a third party—those must be specified.

Reviewer #1: Yes

Reviewer #2: Yes

4. Is the manuscript presented in an intelligible fashion and written in standard English?

PLOS ONE does not copyedit accepted manuscripts, so the language in submitted articles must be clear, correct, and unambiguous. Any typographical or grammatical errors should be corrected at revision, so please note any specific errors here.

Reviewer #1: No

Reviewer #2: Yes

5. Review Comments to the Author

Please use the space provided to explain your answers to the questions above. You may also include additional comments for the author, including concerns about dual publication, research ethics, or publication ethics. (Please upload your review as an attachment if it exceeds 20,000 characters)

Reviewer #1: • The publication by Amare Kassaw1 et al “Incidence and predictors of tuberculosis among HIV-infected children after initiation of antiretroviral therapy in Ethiopia: A systematic review and meta-analysis’, was read and reviewed with great passion and sincerity. I note that this study was registered in PROSPERO on 06/07/2023 with registration number CRD42023439555 as required of systematic reviews.
• The critical appraisal was done using the Joanna Briggs Institute (JBI) quality assessment checklist for cohort studies as seen in the attached S2 document. This implies that high quality studies were considered since JBI is more sensitive than both CASP and ETQS. This however is dependent on the level of expertise since JBI is not user friendly for students.
• The authors do not provide a detailed explanation of eliminated studies and their disqualifying reasons. This could lead to elimination of relevant and useful studies. The authors could use reference manager to avoid deleting duplicates by one category say author’s name or year of publication.
• I note that the review considered only published articles in open access journals and went ahead to acknowledge the authors of the primary articles considered. I also note that no systematic review has ever analyzed or critiqued the used articles.
• The authors used the random effects model to do the meta-analysis which gives weight smaller but relevant studies which isn’t the case for the fixed effects model.
• The article has got a few grammatical errors (especially mixed use of upper & lower cases in the authors’ addresses) which I feel should be harmonized before its publication, either by use of an expert librarian or any other English language expert.
• I confirm that I have read this submission and can say without doubt that it is of an acceptable scientific and ethical standard for publishing in Plos one.

Reviewer #2: The Manuscript meets all the necessary guidelines to satisfy the PLOS ONE criteria for publication. The analysis is performed approriately and presented in an intelligent manner in addition to fulfilling all the other criteria. It is from this basis that I recommend publication of the manuscript.

6. PLOS authors have the option to publish the peer review history of their article ([what does this mean?](https://journals.plos.org/plosone/s/editorial-and-peer-review-process#loc-peer-review-history)). If published, this will include your full peer review and any attached files.


If you choose “no”, your identity will remain anonymous but your review may still be made public.


**Do you want your identity to be public for this peer review?** For information about this choice, including consent withdrawal, please see our [Privacy Policy](https://www.plos.org/privacy-policy).

Reviewer #1: **Yes:** IVAN AHIMBISIBWE

Reviewer #2: **Yes:** Victor Draman Afayo
