# Supplementary material for: Incidence and predictors of tuberculosis among HIV-infected children after initiation of antiretroviral therapy in Ethiopia: A systematic review and meta-analysis
Source: PLoS One. 2024 Jul 5;19(7):e0306651. doi: 10.1371/journal.pone.0306651 (PMC11226042; doi:10.1371/journal.pone.0306651)
Supplement: Attachment — Submitted filename: Response to reviewers.docx [file pone.0306651.s009.docx]

**Response to Reviewers and Academic editor**

**Point by point responses**

We would like to take this opportunity to thank the reviewers and editor for sharing their view and constructive comments. The comments were very important which further improves the quality of our manuscript. The point-by-point responses for each of the comments are provided in the following pages. **Our responses are written in blue font color.**

**#** Journal requirements

1. Please ensure that your manuscript meets PLOS ONE's style requirements, including those for file naming

**Authors’ Response**

We are grateful to this comment of technical relevance. Thus, we have ensured that our manuscript meets PLOS ONE's style requirements, including those for file naming

2. Please review your reference list to ensure that it is complete and correct. If you have cited papers that have been retracted, please include the rationale for doing so in the manuscript text, or remove these references and replace them with relevant current references. Any changes to the reference list should be mentioned in the rebuttal letter that accompanies your revised manuscript. If you need to cite a retracted article, indicate the article’s retracted status in the References list and also include a citation and full reference for the retraction notice.

**Authors’ Response**

The authors are very grateful of these constructive comments. After read the reference lists carefully, we made some correction and addressed the incomplete one. We are sure that we have not cited retracted references, almost all references are recent.

Additional Editor Comments**: #**

Congratulation to come up with this very relevant manuscript of childhood TB among HIV infected children on ART in Ethiopia. It is well narrated, reviewed, analyzed and discussed. Please just attend to a few comments from the one of the revue.

**Authors’ Response**

**Dear editor, really thank you very much for your constructive comments that makes us strive for further relevant works and contribution of our future career in the field.**

**Response to Reviewer #1 comments**

1. The authors do not provide a detailed explanation of eliminated studies and their disqualifying reasons. This could lead to elimination of relevant and useful studies. The authors could use reference manager to avoid deleting duplicates by one category say author’s name or year of publication.

**Authors’ Response**

First of all, we thank you the reviewer for his constructive comments and suggestions. The authors had used EndNote X8 reference manager/citation manager to screen the included studies consciously. After export the search result to EndNote X8 software, the authors used to screen orderly based on the title, abstract, full text, setting, population etc. We worked on carefully to include all the relevant studies. Moreover the authors tried to show the detail of explanation how to screen studies in Figure 1 of page 7.

2. The article has got a few grammatical errors (especially mixed use of upper & lower cases in the authors’ addresses) which I feel should be harmonized before its publication, either by use of an expert librarian or any other English language expert.

**Authors’ Response**

We accepted the comment and correct on the revised manuscript. After we have read carefully through the whole document, we properly addressed the concerned issues.

Reviewer #2:

The Manuscript meets all the necessary guidelines to satisfy the PLOS ONE criteria for publication. The analysis is performed appropriately and presented in an intelligent manner in addition to fulfilling all the other criteria. It is from this basis that I recommend publication of the manuscript

**Authors’ Response**

Really thank you very much.
